# Flow cytometric readout based on Mitotracker Red CMXRos staining of live asexual blood stage malarial parasites reliably assesses antibody dependent cellular inhibition

**DOI:** 10.1186/1475-2875-11-235

**Published:** 2012-07-20

**Authors:** Prajakta S Jogdand, Susheel K Singh, Michael Christiansen, Morten H Dziegiel, Subhash Singh, Michael Theisen

**Affiliations:** 1 Centre for Medical Parasitology at Department of International Health, Immunology and Microbiology, University of Copenhagen, Copenhagen, Denmark; 2 Department of Clinical Biochemistry and Immunology, Statens Serum Institut, Copenhagen, Denmark; 3 H: S Blood Bank KI 2034, Copenhagen University Hospital, Copenhagen, Denmark; 4 Indian Institute of Integrative Medicine, Jammu, India

**Keywords:** *Plasmodium falciparum*, Antibody dependent cellular inhibition assay, Mitotracker Red CMXRos, GLURP

## Abstract

**Background:**

Functional *in vitro* assays could provide insights into the efficacy of malaria vaccine candidates. For estimating the anti-parasite effect induced by a vaccine candidate, an accurate determination of live parasite count is an essential component of most *in vitro* bioassays. Although traditionally parasites are counted microscopically, a faster, more accurate and less subjective method for counting parasites is desirable. In this study mitochondrial dye (Mitotracker Red CMXRos) was used for obtaining reliable live parasite counts through flow cytometry.

**Methods:**

Both asynchronous and tightly synchronized asexual blood stage cultures of *Plasmodium falciparum* were stained with CMXRos and subjected to detection by flow cytometry and fluorescence microscopy. The parasite counts obtained by flow cytometry were compared to standard microscopic counts obtained through examination of Giemsa-stained thin smears. A comparison of the ability of CMXRos to stain live and compromised parasites (induced by either medium starvation or by anti-malarial drug treatment) was carried out. Finally, parasite counts obtained by CMXRos staining through flow cytometry were used to determine specific growth inhibition index (SGI) in an antibody-dependent cellular inhibition (ADCI) assay.

**Results:**

Mitotracker Red CMXRos can reliably detect live intra-erythrocytic stages of *P. falciparum*. Comparison between staining of live with compromised parasites shows that CMXRos predominantly stains live parasites with functional mitochondria. Parasite counts obtained by CMXRos staining and flow cytometry were highly reproducible and can reliably determine the ability of IgG from hyper-immune individuals to inhibit parasite growth in presence of monocytes in ADCI assay. Further, a dose-dependent parasite growth inhibitory effect could be detected for both total IgG purified from hyper-immune sera and affinity purified IgGs against the N-terminal non-repeat region of GLURP in ADCI assays coupled with determination of parasite counts through CMXRos staining and flow cytometry.

**Conclusions:**

A flow cytometry method based on CMXRos staining for detection of live parasite populations has been optimized. This is a rapid and sensitive method with high inter-assay reproducibility which can reliably determine the anti-parasite effect mediated by antibodies in functional *in vitro* assays such as ADCI assay.

## Background

Development of vaccines for malaria remains a high priority in the effort to control malaria worldwide. Blood-stage vaccines are important components of these efforts and functional *in vitro* assays are particularly needed to facilitate the clinical evaluation of candidate vaccines and possibly for future down-selection of vaccine candidates. The antibody-dependent cellular inhibition (ADCI) assay may provide one such tool [1]. Druilhe and co-workers have hypothesized that *Plasmodium falciparum*-specific antibodies can co-operate with human blood monocytes to control parasite multiplication *in vivo* and have accordingly developed the *in vitro* correlate of this immune effector mechanism – the antibody-dependent cellular inhibition (ADCI) assay [2]. Immune-epidemiological studies support the *in vivo* relevance of a monocyte-dependent, antibody-mediated mechanism by showing a correlation between the acquisition of clinical immunity and levels of cytophilic IgG subclasses (IgG1 and IgG3) [3,4]. Cytophilic antibodies opsonize merozoites and activate the monocyte by binding FcγIIa/FcγIIIa present on its cell surface [5,6]. Upon activation, monocytes are thought to release TNF, and other as yet uncharacterized factor(s), that inhibit intra-erythrocytic parasite growth [5,7]. Among the various targets of ADCI-effective antibodies, merozoite surface protein 3 (MSP3) and the glutamate-rich protein (GLURP) have been extensively studied. Affinity-purified IgG to MSP3 and GLURP obtained from endemic sera, have significantly reduced parasite growth in *in vitro* ADCI assay [8,9]. When tested individually in Phase-1 clinical trials in malaria-naïve volunteers, both antigens were found to elicit antibodies capable of mediating parasite killing in *in vitro* ADCI assays [10,11]. These findings have led to the production and clinical testing of a chimeric protein, GMZ2, containing both MSP3 and GLURP domains [12-15]. A robust and high throughput method for assessment of ADCI activity is highly desirable for evaluation of clinical trial samples from large Phase 2 efficacy trials.

Historically, *P. falciparum* growth *in vitro* has been monitored by microscopy, radioactive hypoxanthine uptake [16], and by an enzyme-based method [17]. Radioactive labelling and enzyme-based methods can be problematic in ADCI assays, which include monocytes together with parasitized erythrocytes, as monocytes could contribute to the readout. Microscopic examination of Giemsa-stained slides, therefore, remains the ‘gold standard’ for the quantification of blood-stage malaria parasites in ADCI assays. However, microscopic evaluation is time-consuming and relies on the skills of the microscopists trained perfectly to identify the different parasite stages and to distinguish between live and compromised parasites [18]. These shortcomings are of particular concern, since inter-reader variability gives rise to the common criticism that microscopy is relatively subjective [19,20]. Thus, there is a need for an improved readout for parasite counts in the ADCI assay. Parasite quantification based on flow cytometry has been proposed with the goal of increasing accuracy and reducing subjectivity. Different permeable nucleic acid binding dyes such as Hoechst 33258, 33342 [21], SYBR Green I [22-24], thiazole orange [25], acridine orange [26], ethidium bromide [27], hydroethidine [28,29], SYTO-16 [30], or propidium iodide [31] have been used for enumeration of infected erythrocytes. However, these dyes except possibly hydroethidine, are unable to distinguish between live and compromised parasites as they could stain remnant DNA and/or RNA in compromised parasites. Mitochondrial membrane potential is a key indicator of cellular viability since it reflects metabolic activity and integrity [32]. Dyes which only bind the polarized mitochondrial membrane have been developed to differentiate between live and compromised cells [33]. However, these dyes have inherent problems as rhodamine 123 is highly susceptible to photo-bleaching and exhibits strong photo-induced toxicity, and JC-1 is not only specific for mitochondria [34]. Moreover, both dyes display reversible binding to the polarized membrane leading to potential losses during sample preparation for flow cytometry. Further, rhodamine dyes have adverse effects on mitochondrial respiration [35,36] and do not appear to be strictly dependent on mitochondrial membrane potential for intramitochondrial accumulation. Their use in careful studies on mitochondrial physiology is therefore problematic. Mitotracker Red CMXRos dye has the following advantages: it is intrinsically fluorescent, binds irreversibly to the polarized mitochondrial membrane, and does not require reduction or oxidation for emission of fluorescence [37]. CMXRos dye has an alkylating chloromethyl group, which can react with accessible nucleophiles, including thiol groups of peptides and proteins, to form aldehyde-fixable conjugates. The covalent binding to the polarized inner mitochondrial membrane enhances dye retention during washing and fixation of cells, thereby making this dye particularly suited for flow cytometry and fluorescence microscopy [38]. Of the above-mentioned dyes, hydroethidine [29,39] and rhodamine 123 [40] have previously been used in flow cytometry-based readouts for parasite counts in ADCI assays. In this study, a protocol for flow cytometric evaluation of CMXRos stained parasitized erythrocytes was developed and compared with microscopy. It was concluded that the CMXRos staining-based flow cytometric protocol is a rapid and accurate tool for assessing parasitaemia in the ADCI assay.

## Methods

### Parasite culture and synchronization

The laboratory-adapted *P. falciparum* strain NF54 was cultured as previously described [41] with slight modifications. Briefly, culture was maintained in O^+ve^, human erythrocytes (parasitaemia: 1-10%, haematocrit: 2.85%) in parasite growth medium (PGM) consisting of RPMI 1640 (Lonza, USA) supplemented with 25 mM HEPES, 2 mM L-glutamine, 24 mM NaHCO_3_, 25 μg/ml gentamicin and 10% (v/v) heat-inactivated human AB serum in an atmosphere containing 5% O_2_, 5% CO_2_ and 90% N_2_ at 37 °C. Parasites were synchronized for ring stages by repeated sorbitol treatments [42] and schizonts were enriched by flotation on gelatin [43]. The presence of mycoplasma was monitored regularly by MycoAlert® Detection Kit (Lonza, USA).

### Enumeration of parasitized erythrocytes by microscopy

Parasitaemia was determined by microscopy of standard blood smear. In brief, thin blood smear were prepared on glass slides and fixed with 100% methanol. Smears were stained with filtered 1/10 dilution of Giemsa (Merck Co, Germany) solution in phosphate buffered saline (PBS), incubated for 20 min at room temperature, washed with distilled water, dried, and observed under oil immersion lens (100x). Parasitaemia was determined by counting approximately 5,000 erythrocytes.

### Enumeration of parasitized erythrocytes by flow cytometry and fluoroscence microscopy

The CMXRos dye (8-(4’-chloromethyl) phenyl-2,3,5,6,11,12, 14,15-octahydro-1 H,4 H,10 H,13 H-diquinolizino-8 H-xanthylium chloride) was prepared as per the manufacturer’s recommendation (Invitrogen, USA). CMXRos dye was dissolved in DMSO at a concentration of 1 mM and stored at -20 °C until use. A 5 μM working solution in pre-warmed PGM was prepared directly from the stock solution just prior to staining cultured parasites.

For enumeration of parasitaemia by flow cytometry, approximately 10^6^ cells were harvested by centrifugation in a 96-well, round-bottom plate (Nunc, Denmark) at 2,000 rpm for 5 min, re-suspended in 100 μl of 5 μM CMXRos or 0.5 μM coriphosphine O (Tokyo Chemical Industry Co, Japan) in warm PGM, and incubated at 37 °C for 30 min in the dark. Hydroethidine was used for staining as described earlier [7]. The cells were washed twice with 100 μl of warm PGM to remove unbound dye and re-suspended in 200 μl of warm PGM. A minimum of 3 × 10^4^ events or more than 300 infected erythrocytes were recorded on a Beckman Coulter (cytomics FC500 MPL) and plotted in a two-dimensional scattergram of FL3 and FSC or SSC in the logarithmic display. Bound CMXRos dye was visualized by excitation at 488 nm by an argon ion laser [37] and data were analyzed by FLOWJO Software (Version 7.6.1). Parasitaemia was calculated as the percentage of parasitized erythrocytes in the total number of infected and uninfected erythrocytes. Fluorescence microscopy of infected and non-infected erythrocytes stained with CMXRos dye and DAPI (4’, 6-diamidino-2-phenylindole, dihydrochloride, Invitrogen) was performed as described [44].

### Drug treatment and starvation of *plasmodium falciparum*

A highly synchronized culture of *P. falciparum* NF54 at the mid-trophozoite stage (~4% parasitaemia) was treated with a mixture of atovaquone-proguanil (150 μg/ml) for 30 min. For starvation, a highly synchronized culture of *P. falciparum* NF54 at the mid-trophozoite stage was subjected to temperature changes with minimal quantities of PGM. Briefly, the culture at an initial parasitaemia of 3% was kept at 4 °C for 24 hours, incubated overnight at 37 °C, and transferred again to 4 °C for eight hours. Growth medium was not replenished during this treatment. Both conditions included a control culture, which was not subjected to any of the treatments. Parasitized erythrocytes were enumerated by flow cytometry after staining with CMXRos, coriphosphine O, or hydroethidine. Parasites were also visualized by microscopy after staining with Giemsa before and after the treatment. CMXRos and DAPI pictures were taken immediately after mounting a drop of the stained parasite suspension on a slide.

### Preparation of IgG samples

Total IgG from clinically immune Liberian adults and Danish blood donors (NIG) never exposed to malaria was purified as previously described [9]. Five Liberian plasma samples with high concentrations of anti-malaria IgG1 and IgG3 antibodies as determined by a parasite extract ELISA [45] were pooled (HIG). The concentrations of IgG1-4 in HIG and NIG were 8.8 mg/ml, 2.6 mg/ml, 2.2 mg/ml, and 0.4 mg/ml; and 4.0 mg/ml, 2.4 mg/ml, 0.27 mg/ml, and 0.44 mg/ml, respectively determined as described [46]. Affinity purified IgG against GLURP.R0 were obtained as previously described [9]. Purified IgG was prepared in acetate buffer pH 5.5 with 10% maltose, aliquoted and stored at -20 °C. IgG preparations were dialysed with RPMI before used in ADCI assay.

### Preparation of monocytes and antibody dependent cellular inhibition assay (ADCI)

Peripheral blood mononuclear cells (PBMCs) were isolated from buffy coats of healthy Danish blood donors never exposed to malaria by LymphoPrep (Lonza, USA) and used as a source of monocytes for ADCI. One million PBMCs were added to 96-well, flat-bottom culture plates (Nunc, Roskilde, Denmark) in monocyte medium (5% NHS, 1% glutamine and 1% pencillin-streptomycin in RPMI medium) and incubated for two hours at 37 °C and 5% CO_2_ for adherence of monocytes. After incubation, wells were washed three times with 150 μl PGM thereby separating non-adherent mononuclear cells from attached monocytes (~2 × 10^5^ monocytes per well). It has previously been observed that monocytes from individual donors may either enhance or inhibit *P. falciparum* growth [28,47]. Monocytes from 18 donors were therefore screened for their effect on *P. falciparum* growth *in vitro*. Of these, monocytes from six donors neither enhanced nor inhibited parasite growth in the absence of IgG. Monocytes purified from either fresh or frozen PBMCs from two of these donors, repeatedly gave similar SGI values (~50%) in presence of HIG and were therefore used throughout this study. Adhered monocytes were co-cultured with highly synchronized mature *P. falciparum* NF54 schizonts at a 0.5% parasitaemia and a 2.5% haematocrit. Purified IgG dialyzed against RPMI medium was added to wells at the indicated concentration. Final volume in each well was adjusted to 200 μl with PGM. In addition to test IgGs, the following controls were run simultaneously in every experiment on each plate: (i) parasite culture without monocytes, (ii) culture with monocytes and without IgG, (iii) culture with NIG, (iv) culture with monocytes and NIG, (v) culture with HIG, (vi) culture with monocytes and HIG. At 48 hours and 72 hours an additional 50 μl of PGM was added to each well. At the end of the assay (96 hours), parasitaemia was determined in each well by flow cytometry after staining with CMXRos dye and by Giemsa microscopy. In ADCI, detached monocytes could be distinguished from parasitized erythrocytes after plotting acquired data in a two-dimensional scattergram of FL3 and SSC since SSC separate cell populations according to their granularity. The specific growth inhibitory index (SGI) which estimates the parasite growth inhibition due to the effect of test antibodies (Abs) co-operating with monocytes (MN) was calculated as follows: SGI = 100 × (1 − (% parasitaemia with MN and test Abs/% parasitaemia test Abs)/(% parasitaemia with MN and NIG/% parasitaemia NIG)).

### Statistical analysis

Bland-Altman test was used for assessing agreement between two methods of estimating parasitaemia. Differences in parasitaemia estimated by each dye before and after drug treatment were analysed using two-way ANOVA for overall comparison, followed by the Bonferroni test for pairwise comparisons. All statistical analysis was carried out using GraphPad Prism software version 4.0 (PrismPad Software, USA).

## Results

### Mitotracker Red CMXRos staining can reliably differentiate between uninfected and *plasmodium falciparum* parasitized erythrocytes

**Figure 1:**
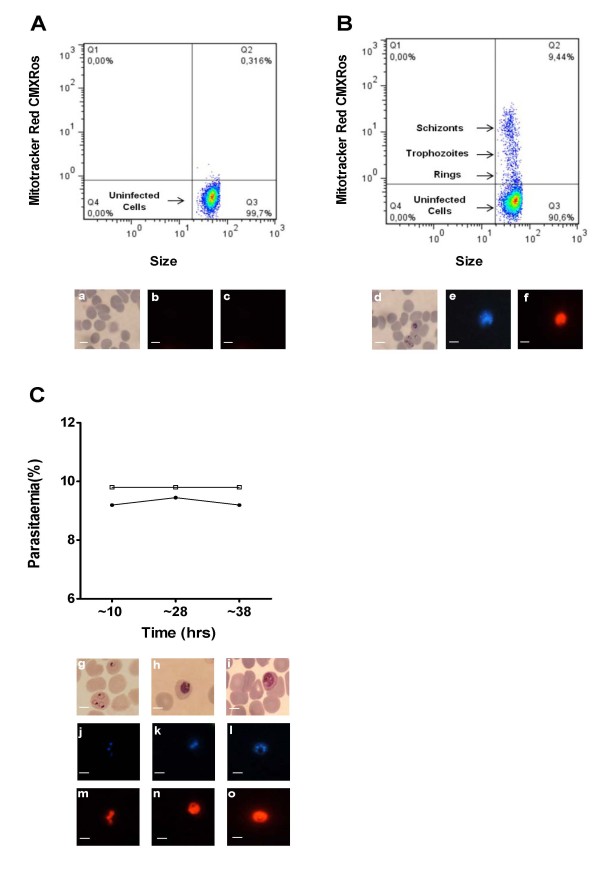
**Flow cytometric analysis of*Plasmodium falciparum*asexual stages stained with CMXRos.** Fluorescence intensity distribution of uninfected erythrocytes (**A**), and asynchronous NF54 *P. falciparum*-infected erythrocytes (**B**). The ordinate shows red fluorescence emission from CMXRos (FL3), and the abscissa displays the forward light scatter signal intensity. Representative microscopic images of Giemsa-stained (panels ‘a’ and ‘d’), DAPI-stained (panels ‘b’ and ‘e’) and Mitotracker Red CMXRos-stained (panels ‘c’ and ‘f’) parasites are shown below the graphs. Time course of intra-erthrocytic *P. falciparum* development (**C**). Samples were from a highly synchronized culture of *P. falciparum* NF54 at ~10 hours, ~28 hours, and ~38 hours post invasion and parasitaemia was determined by flow cytometry after CMXRos staining (closed circles) and by microscopy of Giemsa-stained smears (open squares). Representative images of Giemsa-stained (panels ‘g’, ‘h’ and ‘i’), DAPI -stained (panels ‘j’, ‘k’and ‘l’) and Mitotracker Red CMXRos-stained (panels ‘m’, ‘n’ and ‘o’) parasites are shown below the graph. Scale bars are equal to 5 μm (magnification100x).

**Figure 2:**
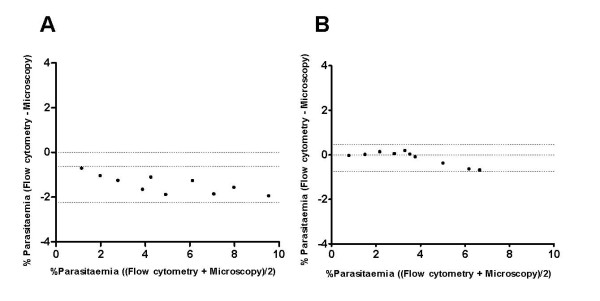
**Agreement between flow cytometric and microscopic readouts of parasitaemia by Bland Altman statistical test.** Differences in % parasitaemia are plotted over the average % parasitaemia measurements. Parasitaemia was determined upon serial dilutions of highly synchronized asexual blood-stage cultures of *P. falciparum* NF54 at the ring stage (**A**) or late trophozoite and schizont stages (**B**) by CMXRos staining and flow cytometry and by microscopy. Dotted lines represent upper and lower 95% limits of agreement between the two techniques. The data is representative from two independent experiment sets.

In order to investigate the usefulness of Mitotracker Red CMXRos for differentiation between uninfected and parasitized erythrocytes, an asynchronous culture of *P. falciparum* NF54 was stained with CMXRos dye. The staining of the cells were observed under fluorescence microscopy and evaluated through flow cytometry. The uninfected erythrocytes did not show any detectable staining when observed through microscopy and appeared as a tightly clustered population in flow cytometry with minimal fluorescence (Figure 1A and panel ‘c’). Infected erythrocytes were identified as cells brightly stained with nuclear stain DAPI under fluorescence microscopy (Figure 1 panel ‘e’). These DAPI positive infected erythrocytes were found to be brightly stained with the CMXRos stain (Figure 1B and panel ‘f’). Further, sorbitol-synchronized parasite populations were used to assess whether the different intra-erythrocytic stages of the parasite could be reliably detected by CMXRos staining. Early rings (~10 hour post-invasion), early trophozoites (~28 hour post-invasion) and late trophozoites (~38 hour post-invasion) were found to be brightly stained with DAPI (Figure 1C panels ‘j’, ‘k’ and ‘l’) and CMXRos dye (Figure 1C panels ‘m’, ‘n’ and ‘o’). Flow cytometry-based evaluation of CMXRos-stained parasite populations at the different stages of intra-erythrocytic development correlated well with the microscopic counts of the Giemsa-stained smears (Figure 1C). The sensitivity of the CMXRos staining-based flow cytometry method for detection of early rings and mature trophozoite populations with different parasitaemia was tested. Determination of parasitaemia by CMXRos-stained flow cytometry and Giemsa-stained microscopic counts for both early rings and mature trophozoites populations ranging between 1-8% parasitaemia were compared in Bland-Altman test to assess the agreement between the two methods (Figure 2A and B). In ring infected erythrocytes, the parasitaemia measured by microscopy was approximately 1.5% higher than by flow cytometry. The 95% limits of agreement for ring and schizont population was (-2.24, -0.61) and (-0.75, 0.48), respectively.

### Mitotracker Red CMXRos staining reliably differentiate between *plasmodium falciparum*-infected erythrocytes containing live and compromised parasites

**Figure 3:**
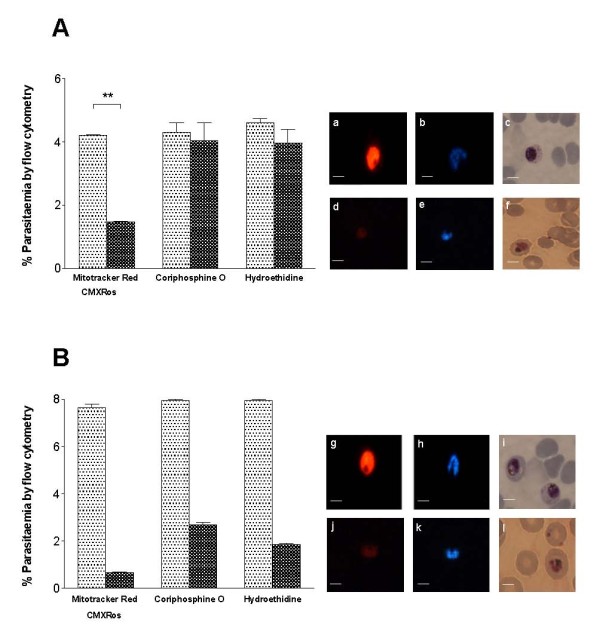
**CMXRos staining differentiates between live and compromised parasites.** Comparative flow cytometric analysis of drug-treated (**A**) and starved (**B**) cultures of *P. falciparum* NF54 stained with CMXRos, coriphosphine O and hydroethidine. Cultures were treated with the two drugs atovaquone-proguanil for 30 min or starved for 48 hours and parasitaemia was determined. Dotted bar represents parasitaemia of the control culture (no treatment) and chequered bar represents parasitaemia after treatment. Error bars show the SEM between duplicate wells of the same sample. Representative data are shown from three independent experiments. Representative images from Mitotracker Red CMXRos (panels ‘a’, ‘d’, ‘g’ and ‘j’), DAPI (panels ‘b’, ‘e’, ‘h’ and ‘k’), and Giemsa (panels ‘c’, ‘f’, ‘i’ and ‘l’) stained live and treated parasites are presented in the panels besides the graph. First row images in each panel represents control culture parasites, while second row has images of parasites taken after the treatment.* *p* < 0.01(Bonferroni pairwise test). Scale bars are equal to 5 μm (magnification100x).

In order to evaluate the ability of CMXRos staining to differentiate between live and compromised states, parasites were exposed to two different growth inhibitory conditions. In the first condition, the binding of CMXRos dye to parasite mitochondria with compromised membrane potential was assessed by treating parasites with a combination of atovaquone and proguanil. There was a significant (p = 0.0094, two-way ANOVA) difference in parasitaemia measured by the three dyes. This difference was due to a significant (p < 0.01, Bonferroni post test) reduction in parasitaemia measured by CMXRos staining but not by coriphosphine O and hydroethidine staining (Figure 3A). This indicates the differential ability of CMXRos dye to detect parasites with intact mitochondrial membrane potential (Figure 3A and panel ‘a’) from those which have compromised mitochondrial membrane potential (Figure 3A and panel ‘d’). In past experiments, hydroethidine has also been used to differentiate between live and dead parasites [28,29]. However, upon treatment with atvoquone-proguanil, hydroethidine staining performed similarly to coriphosphine O and did not show any appreciable decrease in parasitaemia. In the second condition, parasites in the trophozoite stage were exposed to medium starvation and temperature fluctuations for 48 hours. This treatment resulted in highly condensed parasite protoplasm, when observed by microscopy after Giemsa staining, which failed to propagate *in vitro* (Figure 3B panel ‘l’). Medium-starved parasitized erythrocytes also showed marked reduction in CMXRos staining as compared to detection by nucleic acid dye coriphosphine O (Figure 3B). These results strongly suggest that staining by CMXRos detects live and healthy parasite populations characterized by intact mitochondrial membrane potential. Interestingly, hydroethidine-stained compromised parasites stronger than CMXRos but not as efficiently as coriphosphine O, possibly due to some residual activity of the dehydrogenase in the culture.

### Detection of parasites with intact mitochondrial membrane potential through CMXRos staining and flow cytometry can be used to reliably measure ADCI effect

**Figure 4:**
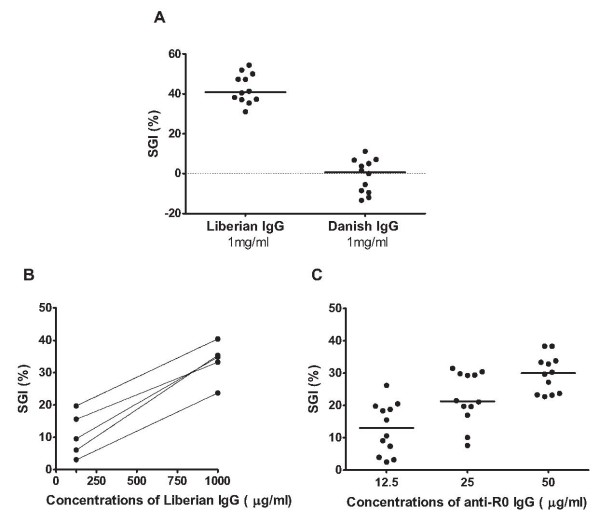
**Reproducibility of ADCI assays applying flow cytometric readout.** SGI values obtained with malaria immune IgG (1 mg/ml) and malaria naïve IgG (1 mg/ml) in 12 independent assays (**A**). Ten assays were performed with monocytes from one donor and two assays were performed with monocytes from another donor. SGI values at two concentrations (125 μg/ml and 1 mg/ml) of malaria immune IgG in five independent experiments (**B**). SGI values obtained at three concentrations (12.5 μg/ml, 25 μg/ml and 50 μg/ml) of affinity-purified anti-GLURP.R0 antibodies (**C**). Parasite growth was monitored by flow cytometry after CMXRos staining. Horizontal lines represent median SGI values.

In order to determine the ADCI effect mediated by antibodies through co-operation with human monocytes and for more accurate calculation of SGI values, parasitaemia was determined by flow cytometry after CMXRos staining, with minimum 30,000 events recorded for each culture sample. This method allowed evaluation of a larger and more homogenous proportion of live, parasitized cells within a culture population without subjective errors associated with classical microscopy counts. The reproducibility of SGI values from 12 independent experiments of purified IgG from hyper-immune Liberians (HIG) and Danish blood donors (NIG) never exposed to malaria were evaluated for ADCI effect (Figure 4A). SGI values for the same 12 experiments with HIG were also calculated by visual inspection of Giemsa-stained thin-blood smears. The coefficient of variation (CV) observed by Giemsa microscopy and Flow cytometry after CMXRos staining was approximately 23.86% and 14.09%, respectively. Both the Liberian and Danish IgG preparations displayed similar profiles for content of cytophilic/non-cytophilic antibodies, indicating that the parasite specificities for these antibody preparations were the major determinants for their parasite killing ability. The reproducibility of SGI effect obtained at two different concentrations of the Liberian IgG was determined. The assay was reproducible with inter-assay coefficient of variation (CV) of 18.22% and 63.19% at 1 mg/ml and 125 μg/ml, respectively; there was a clear dose-dependent effect of the IgG (Figure 4B). Subsequently, affinity purified IgG against GLURP.R0 (non-repeat region), which is a target of naturally occurring antibodies capable of mediating parasite killing through ADCI [9,48] and a component of a leading malaria vaccine candidate GMZ2 [13] in ADCI assays was used to evaluate parasitaemia through CMXRos-stained flow cytometry. As depicted in Figure 4C, a clear-dose dependence effect of the SGI values was observed for the different concentrations of the anti-GLURP.R0 IgG used. Decreasing CVs of 60.87%, 36.24% and 19.32% were found at increasing concentration of GLURP.R0-specific IgG. These results strongly support the usefulness of CMXRos staining for obtaining reliable live parasites counts crucial for assessment of the ADCI effect mediated by antibodies.

## Discussion

Optimized ADCI assay, using Mitotracker Red CMXRos staining and flow cytometry as readout to replace the labour-intensive microscopy was proposed, thus allowing for high throughput and reproducible operator-independent readout method. The main advantage of Mitotracker Red CMXRos dye is that it specifically stains live parasites and binds irreversibly to the polarized mitochondrial membrane, thereby potentially increasing the accuracy of the readout in the ADCI assay. Using monocytes from two pre-selected blood donors and the CMXRos-based flow cytometry readout for obtaining live parasite counts, it has been demonstrated that HIG (1 mg/ml) in 12 independent experiments gives highly reproducible SGI values of approximately 40% with an inter-assay coefficient of variation (CV) of 18.22%. In contrast, IgG from a Danish blood donor never exposed to malaria gave SGI values of approximately 0%. Since the two IgG preparations contain similar IgG subclass profiles, it was concluded that the differences in ADCI activity were due to parasite-specific IgG antibodies in the HIG. In some experiments NIG gave a SGI value below zero. A negative SGI value can be attributed to enhancement of parasite growth, which has also been observed in other ADCI studies [7]. SGI values reflected the concentration of parasite-specific IgG. Decreasing the concentration of hyper-immune IgG decreased the SGI value in a reproducible manner. The reproducibility of the ADCI assay was further evaluated using affinity-purified IgG against GLURP.R0. As seen previously [9], a positive correlation between the concentration of the GLURP.R0-specific IgG and SGI was observed. Interestingly, the inter-assay CV decreases (60.87%, 36.24% and 19.32%) at increasing IgG concentration (12.5 μg/ml, 25 μg/ml, and 50 μg/ml). The reverse relationship between IgG concentration and inter-assay CV conforms to expectations and suggests that higher IgG concentrations gives a more uniform activation of monocytes in the well and a more reproducible parasite killing. Thus, using CMXRos staining-based flow cytometry readout in ADCI assays, it is possible to differentiate between positive and negative samples in a reproducible manner and the SGI value is a reflection of the level of parasite-specific antibodies in the test sample. Apart from the controlled source of monocytes and the operator-independent readout, the choice of the Mitotracker Red CMXRos dye for detection of parasites with intact mitochondrial membrane potential most likely plays a role for the relatively high assay precision.

In ADCI, cross-linking of Fcγ receptors is thought to activate human monocytes to secrete inflammatory cytokines, such as TNF [5], known to compromise mitochondrial membrane potential [49].It was therefore hypothesized that mitochondrial membrane potential dependent dyes are ideal for assessing inhibition of parasite growth in the ADCI assay as they are predicted to efficiently differentiate between live and compromised parasites. Mitotracker Red CMXRos is a cationic dye, which accumulates in the mitochondrial membrane of living cells. In contrast to other mitochondrial dyes such as rhodamine 123 or JC-1, CMXRos binds covalently to the polarized mitochondrial membrane, thereby leading to retention of the dye during washing and fixation, and most likely increased assay reproducibility. The relatively high concentration of Mitotracker Red CMXRos used in this study compared to the concentration used with human cells [50] may be due to the fact that the dye has to traverse multiple host and parasite membranes in order to reach the inner membrane of *P. falciparum* mitochondria. Accordingly, JC-1 has also been used at a similar high concentration for the staining of the *P. falciparum* parasites [51].

To verify the specificity of the flow cytometry method, a parasite culture was treated with the combination drug, atovaquone-proguanil. Atovaquone causes depolarization of mitochondrial membrane potential [52] leading to parasite death, while proguanil has been observed to potentiate the effect of atovaquone [53]. As expected, this treatment resulted in a pronounced reduction in CMXRos staining as visualized by flow cytometry and fluorescence microscopy while these compromised parasites stained strongly with the DNA-dyes coriphosphine O and DAPI. Compromised parasites were also obtained by medium starvation and temperature fluctuations, a method that does not directly target the membrane potential. As for the drug treatment, condensed pycnotic forms of the parasite were visible by Giemsa microscopy. These forms did not stain with CMXRos but stained strongly with coriphosphine O and DAPI, demonstrating that the novel flow cytometric readout can differentiate between live and compromised parasites. This is particularly important for the ADCI assay since the killing factors produced by activated monocytes are known to generate compromised parasites which can be difficult to distinguish from live parasites by microscopy. Interestingly, hydroethidine stained the compromised parasites generated under these experimental conditions, suggesting that CMXRos may be a more sensitive dye for live parasites.

## Conclusions

A new method for enumeration of parasitaemia based on flow cytometry was developed after Mitotracker Red CMXROS staining. Using this method as readout, the ADCI assay is able to differentiate between IgG samples from hyper-immune and malaria-naïve individuals in a highly reproducible manner with an inter-assay coefficient of variation below 20% for the highest concentration of test IgGs. Moreover, the SGI value obtained is a reflection of the level of parasite-specific antibodies in the test sample. This method is suitable for high-throughput analysis of samples from clinical trials and possible for future down-selection of blood-stage vaccine candidates.

## Abbreviations

CMXRos, Mitotracker Red CMXRos; HIG, pool of hyper immunoglobulins; NIG, pool of normal immunoglobulins; GLURP, glutamate-rich protein.

## Competing interests

The authors declare that they have no competing interests.

## Authors' contributions

PSJ, SS and MT conceived the study. PSJ, SKS, MC and MD performed the laboratory work and the statistical analysis. PSJ, SKS, SS and MT wrote the manuscript. All authors have read the manuscript and agree with its contents.
